# Low risk of serological cross-reactivity between dengue and COVID-19

**DOI:** 10.1590/0074-02760200225

**Published:** 2020-08-14

**Authors:** Michele Spinicci, Alessandro Bartoloni, Antonia Mantella, Lorenzo Zammarchi, Gian Maria Rossolini, Alberto Antonelli

**Affiliations:** 1 University of Florence, Department of Experimental and Clinical Medicine, Florence, Italy; 2 Careggi University Hospital, Infectious and Tropical Diseases Unit, Florence, Italy; 3 Careggi University Hospital, Microbiology and Virology Unit, Florence, Italy

**Keywords:** COVID-19, SARS-CoV-2, dengue, antibodies

## Abstract

In the near future, the overlap of Coronavirus disease 2019 (COVID-19) and dengue epidemics is a concrete threat in tropical regions. Co-epidemics of COVID-19 and dengue could be an overwhelming challenge for health systems in low- and middle-income countries. In this work, we investigated potential serological cross-reactions between COVID-19 and dengue patients. Among 32 COVID-19 positive sera, no positive Dengue virus (DENV) IgG/IgM results were observed. On the other hand, one false-positive result was observed among 44 DENV-positive sera tested for COVID-19 antibodies with each of the two rapid tests used. Further data on accuracy of COVID-19 diagnostic test are urgently warranted.

Since December 2019, Coronavirus disease 2019 (COVID-19), caused by severe acute respiratory syndrome coronavirus 2 (SARS-CoV-2), emerged in the international scene as a major public health concern. COVID-19 pandemic is having a disrupting impact on health systems throughout Asia, Europe and America.^1^ At the same time, a large outbreak of dengue is ongoing in Latin American countries, with several deaths being recorded.^2^

In the near future, the overlap of COVID-19 and dengue epidemics is a concrete threat in tropical regions.^3^ ^,^ ^4^ COVID-19 and dengue share several features of clinical and laboratory presentation, and differential diagnosis should rely on specific diagnostic tests.^5^ Recently, two cases of false-positive results by rapid diagnostic test (RDT) for dengue in patients with SARS-CoV-2 infection were reported from Singapore.^6^ If serological cross-reactivity between patients with COVID-19 and dengue will be confirmed, it may result in a high number of misdiagnoses, with dangerous consequences both from the patients and from public health point of view.^7^

In order to further explore this possibility, we investigated potential serological cross-reactions between COVID-19 and dengue patients, by performing commercial assays for detection of anti-Dengue virus (DENV) and anti-SARS-CoV-2 antibodies on sera from well-characterised COVID-19- and dengue-positive patients, respectively.

A total of 32 anonymised sera, obtained from symptomatic patients with COVID-19 diagnosed by positive anti-SARS-CoV-2 IgM/IgG (32/32, tested by 2019-NCOV IgG/IgM Rapid Test Cassette, ScreenItalia, Perugia, Italy) and/or RT-PCR for SARS-CoV-2 on nasopharyngeal swab (26/32), were tested by Dengue Enzyme Linked Immune Assay (ELISA) DENV IgG-IgM (VIRCELL, Granada, Spain). Sera were collected and tested in Italy, at the Careggi University Hospital of Florence, where dengue is not endemic, and previous exposure to DENV was unlikely.

Moreover, 44 anonymised DENV-positive sera, from cases of acute dengue (27 of whom confirmed by NS1 antigen positivity, plus 17 probable diagnosis with only IgM and IgG positivity), were tested by using COVID-19 IgG/IgM Rapid Test Cassette (Orient Gene, Zhejiang, China) and 2019-NCOV IgG/IgM Rapid Test Cassette (ScreenItalia, Perugia, Italy). All DENV-positive sera were collected from travellers, before SARS-CoV-2 emergence.

Among 32 COVID-19 positive sera, no positive DENV IgG/IgM results were observed. On the other hand, one false-positive result was observed among 44 DENV-positive sera tested for COVID-19 antibodies with each method (in one case for IgG and IgM, and in another for IgG only), in two different samples.

Serological test for dengue are known to be affected by cross-reactivity issues in areas were multiple Flavivirus, such as Zika, Yellow fever or Japanese encephalitis virus are circulating. The risk of false-positive results is reduced when dengue IgM/IgG testing is paired with NS1 antigen capture.^8^

According with our results, the concern about false-positive dengue serology in COVID-19 patients could be downsized, at least when an ELISA is used. On the other hand, COVID-19 false-positive results are possible in patients with DENV infection, although at a low rate and variably using different RDTs for COVID-19. It should be also considered that these COVID-19 false-positive results may reflect different aspecific cross-reactivity, not necessarily related to DENV-specific antibodies.^9^ Of course, this is a preliminary study on a limited number of samples, which need to be validated on a wider population.

In low- and middle-income countries, where arboviruses and other common tropical diseases are highly endemic, SARS-CoV-2 spread will represent an additional challenge for clinicians.^10^ ^,^ ^11^ In particular, co-epidemics of COVID-19 and dengue could be an overwhelming situation for health systems.^12^ Further studies are warranted to better elucidate the accuracy of commercially available RDTs for SARS-CoV-2 antibodies detection and their role within the strategy for COVID-19 diagnosis and control.
